# Effect of Pyrazosulfuron-Methyl on the Photosynthetic Characteristics and Antioxidant Systems of Foxtail Millet

**DOI:** 10.3389/fpls.2021.696169

**Published:** 2021-08-05

**Authors:** Ke Ma, Weili Zhang, Liguang Zhang, Xiaoyong He, Yu Fan, Sher Alam, Xiangyang Yuan

**Affiliations:** State Key Laboratory of Sustainable Dryland Agriculture (in Preparation), College of Agronomy, Shanxi Agricultural University, Shanxi, China

**Keywords:** pyrazosulfuron-methyl, foxtail millet, AsA-GSH cycle, photosynthetic system II, herbicide

## Abstract

Foxtail millet (*Setaria Italica* L.) plays a principal role in food security in Africa and Asia, but it is sensitive to a variety of herbicides. This study was performed to clarify whether pyrazosulfuron-methyl can be used in foxtail millet fields and the effect of pyrazosulfuron-methyl on the photosynthetic performance of foxtail millet. Two foxtail millet varieties (Jingu 21 and Zhangzagu 10) were subjected to five doses (0, 15, 30, 60, and 120 g ai ha^−1^) of pyrazosulfuron-methyl in pot and field experiments. The plant height, leaf area, stem diameter, photosynthetic pigment contents, gas exchange parameters, chlorophyll fluorescence parameters, antioxidant enzyme activities, and antioxidant contents at 7 and 15 days after pyrazosulfuron-methyl application, and the yield of foxtail millet were measured. The results suggested that pyrazosulfuron-methyl inhibited the growth of foxtail millet and reduced the photosynthetic pigment contents, photosynthetic rate, and photosynthetic system II activity. Similarly, pyrazosulfuron-methyl decreased the antioxidant enzyme activities and antioxidant contents. These results also indicated that the toxicity of pyrazosulfuron-methyl to foxtail millet was decreased gradually with the extension of time after application; however, the foxtail millet yield was still significantly reduced. Therefore, pyrazosulfuron-methyl is not recommended for application in foxtail millet fields.

## Introduction

Foxtail millet (*Setaria Italica* L.) is a novel model species in C_4_ Gramineae crops (Hu et al., 2018). This species plays a vital role in the adjustment of the crop planting structure in China (Diao, 2007). Many types and high densities of weeds in fields impact the yield and quality of foxtail millet (Zhou et al., 2013). Chemical weeding is one of the most efficient means to control the harmfulness of weeds in modern agricultural production, but few herbicide varieties have been registered for use in foxtail millet fields. Long-term continuous use of the same herbicide will result in weed resistance. Therefore, screening of safe and efficient herbicides is crucial for chemical weeding in the fields of foxtail millet.

Pyrazosulfuron-methyl is a sulfonylurea selective systemic herbicide with a good control effect on broad-leaf weeds and some sedges in rice fields; however, there are few reports on the application of pyrazosulfuron-methyl in foxtail millet fields (Li, 2010). Although the target of sulfonylurea is acetolactate synthase (ALS), secondary effects such as damage to the photosynthetic system and active oxygen scavenging system (Yuan et al., 2013; Guo et al., 2018; Torra et al., 2021), imbalanced sugar metabolism, and blocked transport of photosynthetic products can also inhibit plant growth and cause plant death (Yang et al., 2017). The detection of chlorophyll fluorescence dynamics is a rapid and non-invasive probe for researching plant photosynthetic functions and reflecting the state of crops under herbicide stress (Yuan et al., 2013). Yuan et al. (2013) found that the maximum photochemical efficiency (*Fv/Fm*), apparent electron transport rate (ETR), and photochemical quenching coefficient (*qP*) of *Radix Isatidis* were significantly reduced under the recommended dose of sigma broad. Acetochlor damaged photosynthetic system II (PSII) in maize leaves and blocked photosynthesis (Feng et al., 2021). Once the plant was subjected to stress (herbicide), the system of generating and removing reactive oxygen species (ROS) was destroyed. Under normal conditions, plants can remove excess ROS through the combined action of enzymatic and non-enzymatic antioxidant systems to maintain a steady state of cells (Tibor et al., 2003; Esen et al., 2006). Wang and Zhou (2006) suggested that chlorimuron-ethyl increased the superoxide dismutase (SOD) and peroxidase (POD) activities and the malondialdehyde (MDA) content in wheat. Under nicosulfuron stress, ascorbate peroxidase (APX), glutathione peroxidase (GPX), monodehydroascorbate reductase (MDHAR), dehydroascorbate reductase (DHAR), and glutathione reductase (GR) activities; dehydroascorbate (DHA) and glutathione (GSH) contents; and GSH/oxidized GSH (GSSG) ratios were reduced, and the ascorbate-glutathione cycle (AsA-GSH cycle) was diminished (Wang J. et al., 2018).

Previous studies have shown that different formulations of sulfonylurea herbicides exhibited different safety levels for foxtail millet. Monosulfuron was safe for Zhangzagu 10 and increased its yield (Gao et al., 2015). The recommended dose of tribenuron-methyl reduced the yield and contents of protein and mineral elements of Jingu 21, but it had no significant effect on the yield and quality of Zhangzagu 10 (Ning et al., 2015). Under sigma broad stress, PSII photochemical activity and sugar metabolism were inhibited in plants (Huang et al., 2015; Yang et al., 2017). After rimsulfuron, nicosulfuron, and thifensulfuron-methyl treatments, foxtail millet seedlings remained small and faded to green, their growth stopped, and the seedlings eventually died (Huang et al., 2005). Therefore, the purpose of this study was to investigate the effect of different pyrazosulfuron-methyl doses on (i) plant growth and the photosynthetic performance and (ii) antioxidant enzyme activities and antioxidant contents of foxtail millet.

## Materials and Methods

### Experimental Design

Pyrazosulfuron-methyl (10%, WP) was provided by Dengfeng Kaifeng, Henan Province Jinbo Pesticide Chemical Co., Ltd. The materials used in this experiment are Zhangzagu 10 and Jingu 21. Zhangzagu 10 was supplied by the Zhangjiakou Academy of Agricultural Sciences of Hebei Province, China, and Jingu 21 was provided by the Shanxi Academy of Agricultural Sciences, China.

The field experiment was conducted at the farm of Shanxi Agricultural University, China, in 2018. The study site has a temperate continental climate. The soil is loam (carbonate brown soil,) containing 20.09 g kg^−1^ organic matter, 37.54 mg kg^−1^ alkaline N, 24.5 mg kg^−1^ available P, and 90.03 mg kg^−1^ available K in the 0–20 cm soil layer. Fifty kilograms of compound fertilizer was applied to each 667 m^2^ of the area before sowing. The experiment was arranged in a randomized complete block design with triplicate plots. At the three-leaf stage (21 days after sowing), the plants were treated with 0 (control, CK), 15 (T1), 30 (T2), 60 (T3), and 120 (T4) g ai ha^−1^ pyrazosulfuron-methyl, and the recommended effective dose given by the manufacturer was 30 g ai ha^−1^. The application was performed with a laboratory pot sprayer equipped with a nozzle calibrated to deliver 450 L ha^−1^. The plant height, leaf area, stem diameter, physiological parameters, and photosynthetic and chlorophyll fluorescence of foxtail millet seedlings were recorded at 7 and 15 days after treatment (DAT). The yield and yield components were measured at the maturity stage (138 days after sowing).

The pot experiment was conducted at the Crop Chemistry Control Center of Shanxi Agricultural University, China, in 2018. Foxtail millet seeds were sown in plastic pots (7 × 7 × 8 cm) filled with nutrient soil. The pots were placed in an artificial climate incubator with a photoperiod of 16/8 h (light/dark), temperatures of 25/18°C (light/dark), the illumination intensity of 12,000 xl, and relative humidity of 70 to 80%. The seedlings were thinned to three plants per pot before the three-leaf stage. At the three-leaf stage, five test doses of pyrazosulfuron-methyl were applied using laboratory pot sprayers, and the spraying method was the same as that in the field study. The damage symptoms of seedlings were recorded every day, and the samples were collected 7 and 15 DAT.

### Measurements

#### Plant Height, Stem Diameter, and Leaf Area

The height and length and width of the leaves of the plant were measured with a ruler. The stem diameter was measured with a Vernier caliper. The leaf area was calculated using the following equation (Yuan et al., 2017b):

$$\begin{array}{l} \text{leaf area}=0.75\;\times \text{ leaf length }\times \text{ leaf width} \end{array}$$

#### Yield and Yield Components

After harvesting, the following traits were measured using a ruler, Vernier caliper, and a 10,000 analytical balance (Mettler-Toledo, LLC. Shanghai, China): the ear length, the ear weight, the ear diameter, the ear grain weight, and other traits.

#### Photosynthetic Gas Exchange

The net photosynthetic rate (*Pn*), transpiration rate (*Tr*), stomatal conductance (*Gs*), and intercellular CO_2_ concentration (*Ci*) were measured by a CI-340 portable photosynthesis system (CID Bio-Science, Inc., USA) from 10:00 to 11:00 a.m. The photosynthetically active radiation (PAR) at the leaf surface was ~1,000 ± 50 μmol m^−2^ s^−1^, the temperature of the leaf chamber was 28 ± 2°C, and the ambient CO_2_ concentration was 400 ± 50 μmol mol^−1^.

#### Chlorophyll Fluorescence

The *Fv/Fm*, ETR, *qP*, and non-photochemical quenching coefficient (*qN*) were measured by a miniaturized pulse-amplitude modulated fluorescence analyzer (Mini-PAM, Walz, Effeltrich, Germany) from 9:00 to 10:00 p.m. The applied actinic light had a photosynthetic photon flux density (PPFD) of 500 μmol (photon) m^−2^ s^−1^. The high light flash used to measure saturated fluorescence had a PPFD of 4,000 μmol (photon) m^−2^ s^−1^ and a duration of 0.8 s.

#### Photosynthetic Pigment Contents

The chlorophyll a, chlorophyll b, total chlorophyll, and carotenoid contents were determined according to the method described by Yuan et al. (2017a). The penultimate leaf was cut into small pieces. Samples (0.1 g) were placed in a 15 ml scale test tube along with 10 ml of 96% ethyl alcohol, covered with a rubber stopper, and kept in a dark cabinet for at least 24 h until the leaves turned white. During this period, the tubes were shaken 3 to 4 times. The chlorophyll a, chlorophyll b, and carotenoid concentrations were measured using a UV 2400 UV-visible spectrophotometer (Sunny Heng ping Instrument, LLC. Shanghai, China), and the absorbance was measured at 470, 649, and 665 nm.

$$\begin{array}{l} {\text{C}}_{\text{a}}=13.95\times {\text{A}}_{665}-6.88\times {\text{A}}_{649}\\ {\text{C}}_{\text{b}}=24.96\times {\text{A}}_{649}-7.32\times {\text{A}}_{665}\\ {\text{C}}_{\text{car}}=1,000\times {\text{A}}_{470}-2.05\times {\text{C}}_{\text{a}}-114.8\times {\text{C}}_{\text{b}}/245\\ \text{Pigment content }\left(\text{mg }{\text{g}}^{-1}\;{\text{F}}_{\text{W}}\right)=\text{C}\times {\text{V}}_{\text{T}}\times {\frac{\text{n}}{\text{F}}}_{\text{W}}\times 1,000 \end{array}$$

In the equation, C is the pigment concentration (mg L^−1^), F_W_ is the fresh weight (g), V_T_ is the total volume of the extraction (mL), and n is the dilution ratio.

#### MDA Content and Antioxidant Enzyme Activities

The MDA content and the activities of SOD (EC 1.15.1.1), POD (EC 1.11.1.7), and catalase (CAT) (EC 1.11.1.6) were determined by the thiobarbituric acid (TBA) test, nitro blue tetrazolium (NTB) method, the guaiacol method, and the UV absorption method, respectively (Gao, 2006).

The activity of APX (EC 1.11.1.11) was determined according to the method described by Yoshiyuki and Kozi (1981) with some minor modifications. Fresh leaves (0.1 g) were homogenized in 2 ml 0.5 mol L^−1^ phosphate buffer (pH 7.0) and centrifuged at 10,000 × *g* for 10 min at 4°C. The reaction mixture contained 3 ml of 0.05 mol L^−1^ sodium phosphate buffer (pH 7.0), 0.4 ml of 0.3 mmol L^−1^ EDTA, 1 ml of 0.9 mmol L^−1^ ascorbate, and 0.2 ml of the supernatant. After incubation for 5 min at 25°C, 0.5 mL of 0.25 mmol L^−1^ H_2_O_2_ was added to the above reaction mixture. The decrease in absorbance at 290 nm was measured for 1 min.

The GR (EC 1.6.4.2) activity was determined according to Halliwell and Foyer (1978) with some minor modifications. Fresh leaves (0.1 g) were ground in an ice bath with 2 ml of 1 mol L^−1^ Tris-HCl (pH 7.5) and centrifuged at 13,000 × *g* for 20 min at 4°C. The reaction mixture contained 3 ml of Tris-HCl (pH 7.5), 3 mmol L^−1^ MgCl_2_, 0.5 mmol L^−1^ GSSG, and 0.15 mmol L^−1^ nicotinamide adenine dinucleotide phosphate (NADPH). After incubation for 5 min at 25°C, the supernatant (200 μl) was added to the above reaction mixture. The absorbance was measured at 340 nm and recorded every 30 s for 3 min.

#### Antioxidant Contents

The estimation of AsA/DHA was measured according to the method described by Jiang and Zhang (2001). Fresh foxtail millet leaves (0.1 g) were homogenized in 2 ml of 5% chilled sulfosalicylic acid in an ice bath and centrifuged at 10,000 × *g* for 15 min at 4°C. The supernatant (200 μl) was collected and neutralized by the addition of 24 μl of 1.84 mol L^−1^ triethanolamine, 5 ml of Na-phosphate buffer (pH 7.5, containing 2.5 mmol L^−1^ EDTA), and 50 μl of 10 mmol L^−1^ DTT for 10 min at 25°C. The above mixture was combined with 50 μl of 0.5% n-ethylmaleimide to remove DTT, 200 μl of 10% trichloroacetic acid (TCA), 44% phosphoric acid, 4% 2,2-bipyridine, and 100 μl of 3% FeCl_3_ for 60 min at 40°C. The change in absorbance was recorded at 525 nm. This method was used to measure the total AsA (AsA+DHA) content, and the measurement of AsA content was used per volume of distilled water to replace DTT and n-ethylmaleimide.

The estimation of reduced GSH/GSSG was carried out according to Carlberg and Mannervik (1985). Fresh foxtail millet leaves (0.1 g) were ground in an ice bath with 2 ml of 5% chilled sulfosalicylic acid in an ice bath and centrifuged at 10,000 × *g* for 15 min at 4°C. A 200 μl aliquot of the supernatant was removed and neutralized by the addition of 24 μl of 1.84 mol L^−1^ triethanolamine, 50 μl of 2-vinyl pyridine and 5% chilled sulfosalicylic acid for 60 min at 25°C for GSSG reductase to mask GSH *via* derivatization and to allow for the determination of GSSG alone. The above reaction was mixed with 2.7 ml of 0.05 mol L^−1^ Na-phosphate buffer (pH 7.5, containing 2.5 mmol L^−1^ EDTA), 20 μl of 0.01 mmol L^−1^ NADPH, and 80 μl of 12.5 mmol L^−1^ 5,5′-dithiobis-(2-nitrobenzoic acid) (DTNB) for 10 min at 25°C. A 20 μl aliquot of GR (50 U mL^−1^) was added, and the change in absorbance at 412 nm was monitored for 3 min. This method was used to measure the GSSG content, and the measurement of total GSH (GSSG+GSH) was used in isopycnic distilled water to replace 2-vinylpyridine.

### Statistical Analysis

Statistical data were analyzed using Microsoft Office Excel 2010 and Data Processing System (DPS 7.05). Duncan's test was used to determine the significant differences among the treatments at the same time and variety at the significance level of *P* ≤ 0.05.

## Results

### Effects of Pyrazosulfuron-Methyl on Plant Height, Stem Diameter, and Leaf Area of Foxtail Millet Seedlings

Under pyrazosulfuron-methyl stress, the plant height, leaf area, and stem diameter of foxtail millet were significantly decreased (Table 1). After exposing the seedlings to the recommended dose of pyrazosulfuron-methyl for 7 days, the plant height, leaf area, and stem diameter of Jingu 21 and Zhangzagu 10 seedlings were significantly decreased by 39, 30, and 12% and 40, 30, and 12%, respectively, while 15 DAT with pyrazosulfuron-methyl, the plant height, leaf area, and stem diameter decreased by 28, 16, and 13% and 40, 35, and 27% for Jingu 21 and Zhangzagu 10 seedlings, respectively. Therefore, at the initial stage of application, the recommended dose of pyrazosulfuron-methyl had severe phytotoxicity to both foxtail millet varieties, but the phytotoxicity gradually decreased over time, and the relief efficiency of Jingu 21 was slightly stronger than that of Zhangzagu 10.

**Table 1:** Effects of pyrazosulfuron-methyl on the plant height, stem diameter, and leaf area of foxtail millet seedlings.

**Varieties**	**Treatments (g ai ha^**−1**^)**	**Plant height (cm)**		**Leaf area (cm** ^****2****^ **)**		**Stem diameter (mm)**	
		**7 DAT**	**15 DAT**	**7 DAT**	**15 DAT**	**7 DAT**	**15 DAT**
Jingu 21	CK	76.18 ± 5.38 a	106.88 ± 3.92 a	99.06 ± 7.17 a	129.38 ± 3.60 a	8.57 ± 0.81 a	10.88 ± 1.63 a
	T1	48.79 ± 4.24 b	86.42 ± 9.89 b	73.05 ± 3.75 b	113.77 ± 3.79 b	7.34 ± 0.76 b	9.19 ± 0.92 b
	T2	46.70 ± 3.92 b	76.50 ± 14.12 bc	68.93 ± 3.33 b	108.17 ± 8.02 b	7.50 ± 0.90 b	9.49 ± 0.87 b
	T3	46.84 ± 3.17 b	67.42 ± 16.90 c	58.96 ± 5.96 c	99.98 ± 6.33 c	7.25 ± 0.44 b	9.24 ± 0.84 b
	T4	46.07 ± 3.66 b	65.94 ± 18.65 c	58.82 ± 5.02 c	90.47 ± 7.29 d	6.97 ± 0.74 b	8.95 ± 0.74 b
Zhangzagu 10	CK	72.26 ± 5.45 a	98.50 ± 10.20 a	92.88 ± 4.82 a	116.38 ± 4.23 a	8.45 ± 0.53 a	11.37 ± 0.85 a
	T1	45.96 ± 5.45 b	62.91 ± 15.28 b	67.95 ± 4.23 b	80.87 ± 4.35 b	8.26 ± 0.54 a	8.45 ± 1.42 b
	T2	43.19 ± 4.54 bc	59.44 ± 18.51 b	64.69 ± 4.17 bc	75.54 ± 3.29 c	7.41 ± 0.95 b	8.28 ± 1.46 b
	T3	41.88 ± 3.35 bc	53.48 ± 10.60 bc	60.52 ± 3.68 c	70.65 ± 3.35 d	7.42 ± 0.98 b	8.33 ± 0.68 b
	T4	40.37 ± 3.51 c	44.69 ± 8.85 c	51.30 ± 4.88 d	61.13 ± 1.54 e	6.50 ± 0.84 c	8.20 ± 1.48 b

*Different letters indicate the significant differences at P ≤ 0.05 among the different treatments at the same time and variety*.

### Effects of Pyrazosulfuron-Methyl on the Photosynthetic Pigment Contents of Foxtail Millet Seedlings

As shown in Table 2, the contents of chlorophyll a, chlorophyll b, total chlorophyll, and carotenoids in the leaves of foxtail millet declined with increasing pyrazosulfuron-methyl dose. Apart from the T1 treatment, the total chlorophyll content of foxtail millet in all other treatments was significantly decreased compared with that of CK at 7 and 15 DAT, but the carotenoid content was not significantly different from that of CK.

**Table 2:** Effects of pyrazosulfuron-methyl on photosynthetic pigment contents of foxtail millet seedlings.

**Varieties**	**DAT**	**Treatments**	**Chlorophyll a content**	**Chlorophyll b content**	**Carotenoid content**	**Chlorophyll content**
		**(g ai ha^**−1**^)**	**(mg g^**−1**^ F_**W**_)**	**(mg g^**−1**^ F_**W**_)**	**(mg g^**−1**^ F_**W**_)**	**(mg g^**−1**^ F_**W**_)**
Jingu 21	7	CK	1.77 ± 0.05 a	0.53 ± 0.08 a	0.32 ± 0.01 a	2.31 ± 0.10a
		T1	1.67 ± 0.10 ab	0.51 ± 0.05 ab	0.31 ± 0.01 a	2.18 ± 0.08 ab
		T2	1.54 ± 0.11 bc	0.45 ± 0.14 ab	0.30 ± 0.02 a	1.99 ± 0.25 bc
		T3	1.51 ± 0.07 bc	0.37 ± 0.02 b	0.31 ± 0.01 a	1.88 ± 0.09c
		T4	1.44 ± 0.17 c	0.37 ± 0.04 b	0.30 ± 0.03 a	1.81 ± 0.21c
	15	CK	1.72 ± 0.03 a	0.62 ± 0.05 a	0.27 ± 0.02 a	2.34 ± 0.06a
		T1	1.62 ± 0.11 ab	0.54 ± 0.01 ab	0.27 ± 0.01 a	2.16 ± 0.11 ab
		T2	1.62 ± 0.08 ab	0.52 ± 0.06 b	0.25 ± 0.03 a	2.14 ± 0.09 b
		T3	1.54 ± 0.11 b	0.49 ± 0.06 b	0.25 ± 0.02 a	2.03 ± 0.17 bc
		T4	1.48 ± 0.03 b	0.46 ± 0.03 b	0.26 ± 0.02 a	1.94 ± 0.06c
Zhangzagu 10	7	CK	1.87 ± 0.09 a	0.70 ± 0.09 a	0.28 ± 0.05 a	2.57 ± 0.16a
		T1	1.73 ± 0.08 ab	0.56 ± 0.12 ab	0.32 ± 0.02 a	2.29 ± 0.18 ab
		T2	1.58 ± 0.12 bc	0.44 ± 0.03 bc	0.31 ± 0.04 a	2.02 ± 0.09 bc
		T3	1.53 ± 0.09 c	0.41 ± 0.03 bc	0.29 ± 0.03 a	1.94 ± 0.12c
		T4	1.51 ± 0.12 c	0.36 ± 0.08 c	0.30 ± 0.02 a	1.87 ± 0.20c
	15	CK	1.58 ± 0.11 a	0.52 ± 0.05 a	0.27 ± 0.01 a	2.10 ± 0.16a
		T1	1.56 ± 0.06 a	0.50 ± 0.04 ab	0.26 ± 0.01 a	2.06 ± 0.06 ab
		T2	1.43 ± 0.13 ab	0.42 ± 0.05 bc	0.25 ± 0.04 a	1.85 ± 0.18 bc
		T3	1.36 ± 0.05 b	0.40 ± 0.03 c	0.25 ± 0.03 a	1.76 ± 0.07c
		T4	1.34 ± 0.06 b	0.39 ± 0.05 c	0.25 ± 0.01 a	1.73 ± 0.11c

*Different letters indicate the significant differences at P ≤ 0.05 among the different treatments at the same time and variety*.

### Effects of Pyrazosulfuron-Methyl on Gas Exchange Parameters of Foxtail Millet Seedlings

**Figure 1:**
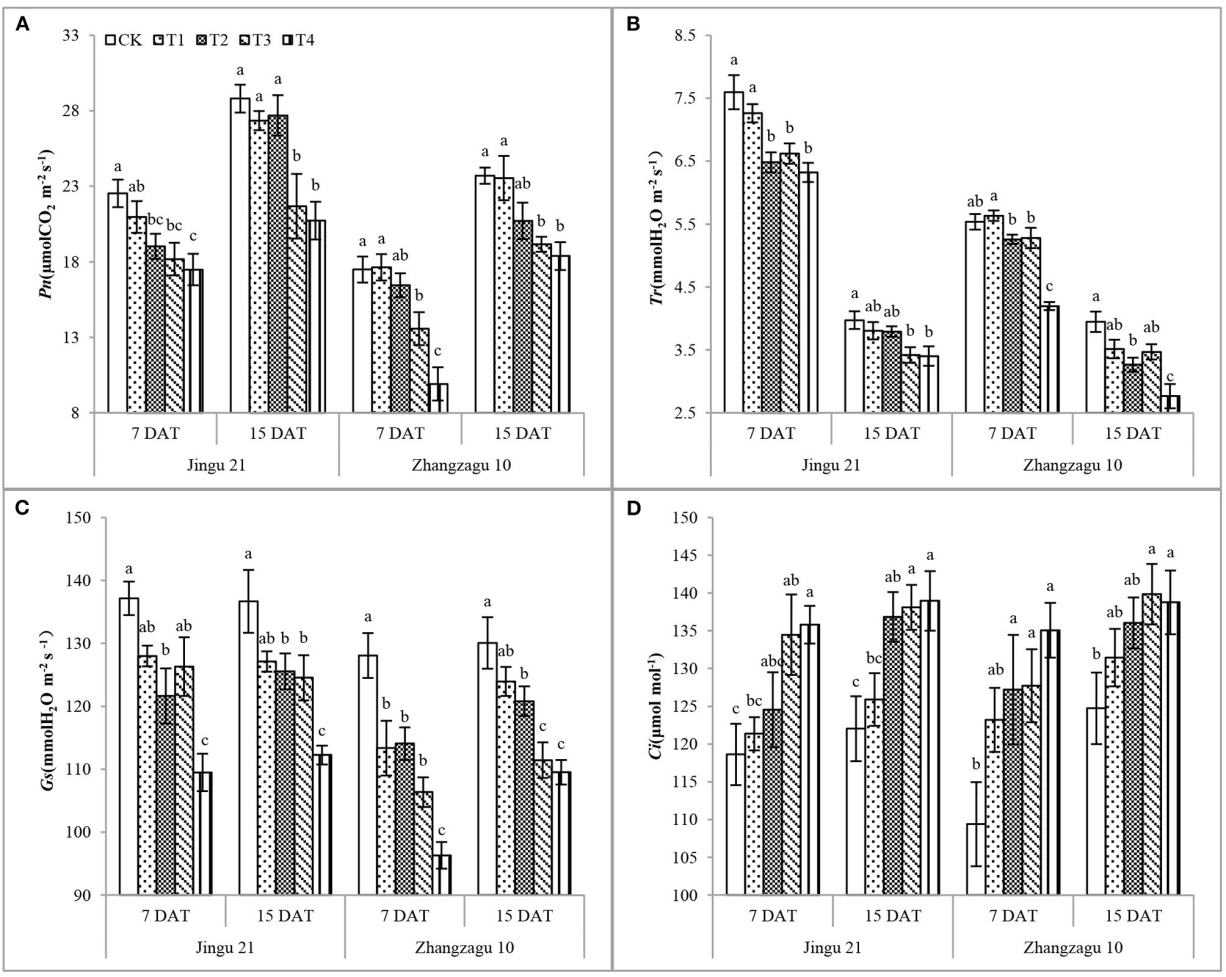
Effects of pyrazosulfuron-methyl on gas exchange parameters [net photosynthetic rate, *Pn* **(A)**, transpiration rate*, Tr* **(B)**, stomatal conductance, *Gs* **(C)**, intercellular CO_2_ concentration*, Ci* **(D)**] of foxtail millet seedlings. CK: water treatment as a control; T1–T4: 15, 30, 60, and 120 g ai ha^−1^ pyrazosulfuron-methyl treatment. Different letters indicate the significant differences at *P* ≤ 0.05 among the different treatments at the same time and variety.

After 7 days of pyrazosulfuron-methyl application at the recommended dose, the *Pn* (Figure 1A), *Tr* (Figure 1B), and *Gs* (Figure 1C) of Jingu 21 seedlings decreased by 16, 15, and 11%, respectively, compared with CK, but the *Ci* (Figure 1D) of Jingu 21 was not significantly different; the *Tr* and *Gs* of Zhangzagu 10 seedlings decreased by 5 and 11%, respectively, and the *Ci* increased by 9%, compared with CK, and the *Pn* showed no significant difference. During 15 DAT, the gas exchange parameters of foxtail millet seedlings showed varying degrees of recovery. After high-dose pyrazosulfuron-methyl treatment (T3, T4), the gas exchange parameters of the foxtail millet seedlings were significantly different from those of the CK seedlings. According to these results, the effects of pyrazosulfuron-methyl on the gas exchange parameters of Zhangzagu 10 were higher than those of Jingu 21. There was no obvious relief with the progression of the fertility process.

### Effects of Pyrazosulfuron-Methyl on Chlorophyll Fluorescence in Foxtail Millet Seedlings

**Figure 2:**
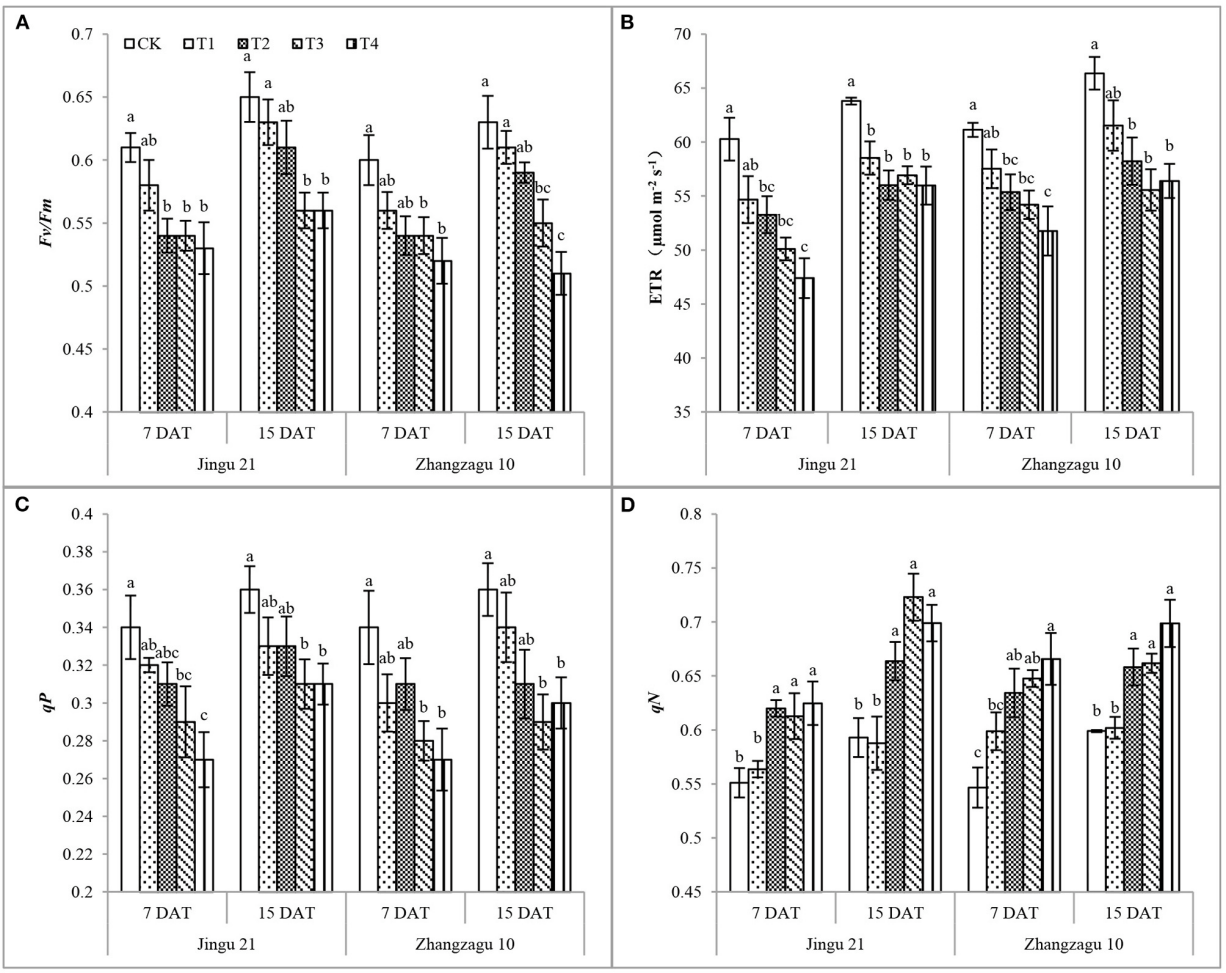
Effects of pyrazosulfuron-methyl on chlorophyll fluorescence [photochemical efficiency*, Fv/Fm* **(A)**, electron transport rate, ETR **(B)**, photochemical quenching coefficient, *qP* **(C)**, non-photochemical quenching coefficient, *qN* **(D)**] of foxtail millet seedlings. CK: water treatment as a control; T1–T4: 15, 30, 60, and 120 g ai ha^−1^ pyrazosulfuron-methyl treatment. Different letters indicate the significant differences at *P* ≤ 0.05 among the different treatments at the same time and variety.

As shown in Figures 2A–D, with increasing pyrazosulfuron-methyl dose, the *Fv/Fm*, ETR, and *qP* of the Jingu 21 and Zhangzagu 10 varieties showed a decreasing trend, while *qN* showed an increasing trend. After 7 days of exposure to pyrazosulfuron-methyl, the *Fv/Fm*, ETR, and *qP* of Jingu 21 were significantly decreased compared with those of CK, while *qN* was significantly increased. After exposing the seedlings to T2 for 15 days, the *Fv/Fm* and *qP* were recovered to the CK level, the ETR was significantly decreased by 12%, whereas *qN* was significantly increased by 12%. The chlorophyll fluorescence characteristics of Jingu 21 were still significantly different from those of CK after T3 and T4 treatments. At 7 and 15 DAT, the ETR of Zhangzagu 10 in T2 was significantly reduced by 9 and 12%, respectively, while *qN* significantly increased by 15 and 10% compared with CK. The chlorophyll fluorescence characteristics of Zhangzagu 10 seedlings always had a significant difference compared with CK after high-dose pyrazosulfuron-methyl treatment (T3, T4).

### Effects of Pyrazosulfuron-Methyl on the MDA Content and SOD, POD, CAT, APX, and GR Activities of Foxtail Millet Seedlings

**Figure 3:**
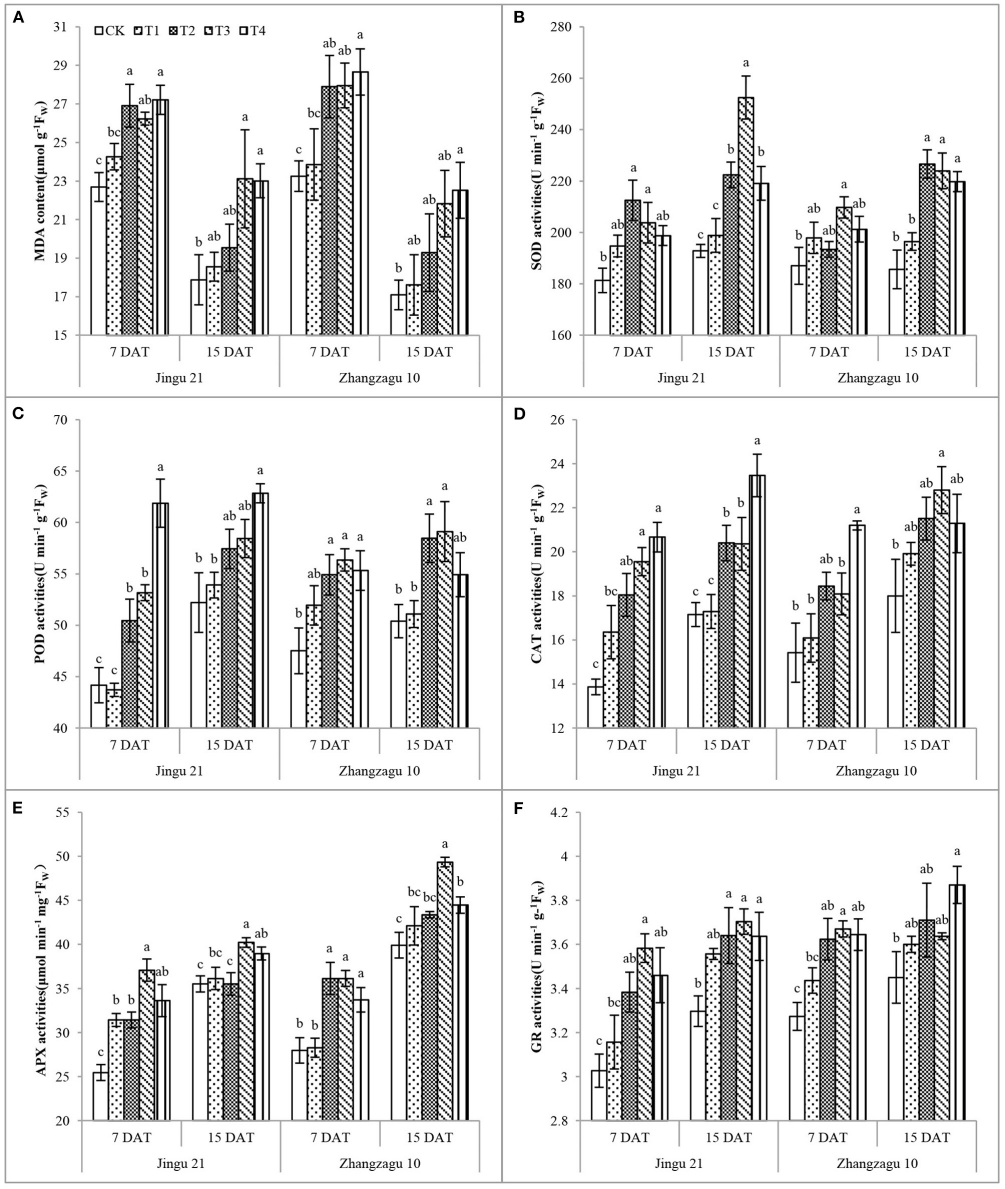
Effects of pyrazosulfuron-methyl on malondialdehyde, MDA content **(A)**; superoxide dismutase, SOD **(B)**; peroxidase, POD **(C)**; catalase, CAT **(D)**; ascorbate peroxidase, APX **(E)** and glutathione reductase, GR **(F)** activities of foxtail millet seedlings. CK: water treatment as a control; T1–T4: 15, 30, 60, and 120 g ai ha^−1^ pyrazosulfuron-methyl treatment. Different letters indicate the significant differences at *P* ≤ 0.05 among the different treatments at the same time and variety.

With increasing pyrazosulfuron-methyl dose, the MDA content and SOD, POD, CAT, APX, and GR activities increased to different degrees (Figure 3). Apart from that of the T1 treatment, the MDA content of foxtail millet in all other treatments was significantly increased compared with the control after 7 days. The MDA content increased by 29% in the T3 treatment group and increased by 29% in the T4 treatment group; the content of MDA in the Zhangzagu 10 subjected to T2 and T3 treatments had no significant difference compared with the CK group, but it significantly increased by 32% at the T4 dose.

At 7 DAT, the highest SOD activity of Jingu 21 and Zhangzagu 10 was observed at T2 and T3, and it was significantly increased by 17 and 12% compared with that of CK. Apart from those of the T1 treatment, the POD, APX, and GR activities of foxtail millet in all other treatments were significantly increased compared with those in CK after 7 days, and there was no obvious relief with the progression of the fertility process. Under T2, T3, and T4 treatments, the CAT activity of Jingu 21 was significantly increased by 30, 41, and 49% and by 19, 19, and 37% after 7 and 15 days, respectively; the CAT activity of Zhangzagu 10 was highest in the T4 treatment after 7 days and in the T3 treatment after 15 days, which were significantly increased by 37 and 27% compared with CK treatment, respectively.

### Effects of Pyrazosulfuron-Methyl on the AsA, DHA, Total AsA Contents, and AsA/DHA Value of Foxtail Millet Seedlings

Different doses of pyrazosulfuron-methyl had different effects on the AsA, DHA, total AsA contents, and AsA/DHA value of foxtail millet leaves (Table 3). The contents of AsA, DHA, and total AsA increased with increasing pyrazosulfuron-methyl dose. After exposing the seedlings to the recommended dose of pyrazosulfuron-methyl, the AsA, total AsA contents, and AsA/DHA value of Jingu 21 were significantly increased by 47, 24, and 83% at 7 DAT, respectively; after 15 days, the AsA content was recovered to the CK level, while the total AsA content and AsA/DHA value were still significantly higher than those in the CK group. In the T1, T2, and T3 groups, the contents of AsA, DHA, and total AsA of Zhangzagu 10 were significantly higher than those of CK at 7 and 15 DAT, and the DHA content was not significantly different from that of CK.

**Table 3:** Effects of pyrazosulfuron-methyl on the ascorbate (AsA), dehydroascorbate (DHA), total AsA contents, and AsA/DHA values of foxtail millet seedlings.

**Varieties**	**DAT**	**Treatments**	**AsA content**	**DHA content**	**Total AsA content**	**AsA/DHA**
		**(g ai ha^**−1**^)**	**(μmol g^**−1**^ F_**W**_)**	**(μmol g^**−1**^ F_**W**_)**	**(μmol g^**−1**^ F_**W**_)**	
Jingu 21	7	CK	1.10 ± 0.22 c	0.74 ± 0.13 b	1.83 ± 0.35 d	1.49 ± 0.03 b
		T1	1.45 ± 0.04 b	0.43 ± 0.09 c	1.88 ± 0.13 cd	3.42 ± 0.59 a
		T2	1.62 ± 0.04 ab	0.65 ± 0.22 bc	2.27 ± 0.17 bc	2.72 ± 1.03 a
		T3	1.79 ± 0.22 a	0.65 ± 0.04 bc	2.44 ± 0.17 b	2.78 ± 0.52 a
		T4	1.49 ± 0.17 ab	1.43 ± 0.04 a	2.92 ± 0.22 a	1.04 ± 0.09 b
	15	CK	2.01 ± 0.09 b	0.22 ± 0.04 d	2.22 ± 0.13 c	9.48 ± 1.53 a
		T1	2.18 ± 0.35 b	0.61 ± 0.17 b	2.79 ± 0.17 b	3.93 ± 1.77 b
		T2	2.44 ± 0.26 ab	0.52 ± 0.09 bc	2.96 ± 0.17 ab	4.85 ± 1.33 b
		T3	2.92 ± 0.30 a	0.35 ± 0.09 cd	3.26 ± 0.22 a	8.96 ± 3.21 a
		T4	2.22 ± 0.30 b	0.91 ± 0.13 a	3.13 ± 0.17 a	2.51 ± 0.70 b
Zhangzagu 10	7	CK	1.53 ± 0.04 d	0.26 ± 0.09 c	1.79 ± 0.13 d	6.35 ± 2.06 a
		T1	1.79 ± 0.13 c	0.74 ± 0.13 ab	2.53 ± 0.26 c	2.47 ± 0.26 b
		T2	2.40 ± 0.13 b	0.74 ± 0.30 ab	3.13 ± 0.17 b	3.76 ± 1.88 b
		T3	2.48 ± 0.13 b	1.00 ± 0.22 a	3.48 ± 0.09 a	2.60 ± 0.72 b
		T4	2.74 ± 0.04 a	0.61 ± 0.09 b	3.35 ± 0.13 ab	4.59 ± 0.59 ab
	15	CK	1.45 ± 0.22 c	0.48 ± 0.04 b	1.92 ± 0.17 d	3.08 ± 0.74 a
		T1	2.18 ± 0.09 b	0.48 ± 0.04 b	2.66 ± 0.13 c	4.60 ± 0.24 a
		T2	2.27 ± 0.09 ab	0.69 ± 0.17 b	2.96 ± 0.09 b	3.44 ± 1.01 a
		T3	2.35 ± 0.26 ab	0.87 ± 0.43 ab	3.22 ± 0.17 b	3.46 ± 2.31 a
		T4	2.57 ± 0.13 a	1.21 ± 0.35 a	3.78 ± 0.22 a	2.27 ± 0.79 a

*Different letters indicate the significant differences at P ≤ 0.05 among the different treatments at the same time and variety*.

### Effects of Pyrazosulfuron-Methyl on the GSH, GSSG, Total GSH Contents, and GSH/GSSG Values of Foxtail Millet Seedlings

With increasing pyrazosulfuron-methyl dose, the GSH and total GSH contents of foxtail millet increased, and the GSSG content increased and subsequently decreased (Table 4). At 7 and 15 DAT, the GSSG content of Jingu 21 was not significantly different from that of CK in all treatments; the GSH and total GSH contents significantly increased under the T2, T3, and T4 treatments. At 7 DAT, the GSH/GSSG values of Jingu 21 significantly increased by 11% under T4 treatment, while 15 days later, it recovered to the CK level. The GSH, GSSG, and total GSH contents of Zhangzagu 10 significantly increased in all treatments at 7 DAT compared with those in CK, and the GSH/GSSG values significantly decreased by 57, 41, and 59% under T1, T2, and T3 treatments, respectively; at 15 DAT, the GSH and total GSH contents were significantly higher in all treatment than in CK, and the content of GSSG increased significantly in T4 but was not significantly different from that in CK under other treatments; furthermore, at 15 DAT, the GSH/GSSG values were recovered to the CK level in all treatments.

**Table 4:** Effects of pyrazosulfuron-methyl on the glutathione (GSH), GSH/oxidized GSH (GSSG), total GSH contents, and GSH/GSSG values of foxtail millet seedlings.

**Varieties**	**DAT**	**Treatments**	**GSH content**	**GSSG content**	**Total GSH content**	**GSH/GSSG**
		**(g ai ha^**−1**^)**	**(μmol g^**−1**^ F_**W**_)**	**(μmol g^**−1**^ F_**W**_)**	**(μmol g^**−1**^ F_**W**_)**	
Jingu 21	7	CK	5.40 ± 0.01 c	0.60 ± 0.01 ab	6.00 ± 0.02 b	9.00 ± 0.12 b
		T1	5.41 ± 0.01 c	0.61 ± 0.00 a	6.02 ± 0.02 b	8.88 ± 0.02 b
		T2	5.46 ± 0.01 b	0.60 ± 0.04 a	6.06 ± 0.03 a	9.09 ± 0.62 b
		T3	5.46 ± 0.04 b	0.62 ± 0.04 a	6.08 ± 0.01 a	8.87 ± 0.66 b
		T4	5.52 ± 0.02 a	0.55 ± 0.03 b	6.07 ± 0.01 a	10.03 ± 0.65 a
	15	CK	5.52 ± 0.02 c	0.59 ± 0.00 ab	6.11 ± 0.02 d	9.33 ± 0.08 ab
		T1	5.57 ± 0.02 b	0.57 ± 0.01 b	6.14 ± 0.02 cd	9.69 ± 0.10 a
		T2	5.57 ± 0.01 b	0.62 ± 0.04 a	6.20 ± 0.03 ab	8.97 ± 0.61 b
		T3	5.61 ± 0.00 a	0.61 ± 0.00 ab	6.22 ± 0.01 a	9.26 ± 0.04 ab
		T4	5.57 ± 0.01 b	0.59 ± 0.01 b	6.16 ± 0.02 bc	9.53 ± 0.08 a
Zhangzagu 10	7	CK	1.53 ± 0.04 d	0.26 ± 0.09 c	1.79 ± 0.13 d	6.35 ± 2.06 a
		T1	1.79 ± 0.13 c	0.74 ± 0.13 ab	2.53 ± 0.26 c	2.47 ± 0.26 b
		T2	2.40 ± 0.13 b	0.74 ± 0.30 ab	3.13 ± 0.17 b	3.76 ± 1.88 b
		T3	2.48 ± 0.13 b	1.00 ± 0.22 a	3.48 ± 0.09 a	2.60 ± 0.72 b
		T4	2.74 ± 0.04 a	0.61 ± 0.09 b	3.35 ± 0.13 ab	4.59 ± 0.59 ab
	15	CK	1.45 ± 0.22 c	0.48 ± 0.04 b	1.92 ± 0.17 d	3.08 ± 0.74 a
		T1	2.18 ± 0.09 b	0.48 ± 0.04 b	2.66 ± 0.13 c	4.60 ± 0.24 a
		T2	2.27 ± 0.09 ab	0.69 ± 0.17 b	2.96 ± 0.09 b	3.44 ± 1.01 a
		T3	2.35 ± 0.26 ab	0.87 ± 0.43 ab	3.22 ± 0.17 b	3.46 ± 2.31 a
		T4	2.57 ± 0.13 a	1.21 ± 0.35 a	3.78 ± 0.22 a	2.27 ± 0.79 a

*Different letters indicate the significant differences at P ≤ 0.05 among the different treatments at the same time and variety*.

### Effects of Pyrazosulfuron-Methyl on the Yield and Yield Components of Foxtail Millet

Under pyrazosulfuron-methyl stress, the ear length, ear diameter, spike weight, grain weight, ear yardage, 1,000-grain weight, the number of ears, and yield of Jingu 21 and Zhangzagu 10 were all decreased to varying degrees (Table 5). Under the T1 treatment, the yield of Jingu 21 was significantly reduced by 9% compared with the CK, while the difference between Zhangzagu 10 and the CK was not significant. When treated at T2, T3, and T4, the yields of Jingu 21 and Zhangzagu 10 were significantly reduced by 16, 35, and 51% and 28, 40, and 51%, respectively.

**Table 5:** Effects of pyrazosulfuron-methyl on the yield and yield components of foxtail millet.

**Varieties**	**Treatments**	**Ear length**	**Ear diameter**	**Spike weight**	**Grain weight**	**Ear yardage**	**1,000-grain**	**Number of**	**Yield**
	**(g ai ha^**−1**^)**	**(cm)**	**(mm)**	**(g)**	**(g)**		**weight**	**ears**	**(kg 667m^**−2**^)**
							**(g)**	**(10^**4**^ ha^**−1**^)**	
Jingu 21	CK	25.61 ± 2.27 a	32.76 ± 2.87 a	34.55 ± 1.76 a	27.87 ± 1.14 a	111.89 ± 7.85 a	3.12 ± 0.34 a	28.67 ± 1.53 a	533.50 ± 14.58 a
	T1	25.47 ± 2.36 a	32.93 ± 2.98 a	34.38 ± 2.50 a	26.75 ± 1.34 a	110.33 ± 6.25 a	3.09 ± 0.22 a	27.33 ± 1.15 a	488.12 ± 12.12 b
	T2	24.79 ± 2.15 a	31.82 ± 3.60 a	32.23 ± 1.81 ab	24.94 ± 1.90 b	106.11 ± 4.99 ab	3.08 ± 0.29 a	27.00 ± 2.00 a	449.63 ± 14.72 c
	T3	24.42 ± 2.07 a	29.68 ± 3.34 ab	31.77 ± 1.94 b	23.21 ± 1.83 c	101.22 ± 6.55 b	3.04 ± 0.19 a	22.67 ± 2.52 b	348.88 ± 5.81 d
	T4	24.13 ± 1.51 a	27.85 ± 4.42 b	28.73 ± 3.55 c	24.98 ± 1.38 b	100.67 ± 8.99 b	3.04 ± 0.09 a	15.67 ± 1.53 c	261.08 ± 8.83 e
Zhangzagu 10	CK	30.27 ± 1.62 ab	34.30 ± 1.53 a	31.19 ± 2.14 a	24.99 ± 2.19 a	115.44 ± 7.72 a	3.25 ± 0.17 a	35.00 ± 1.00 a	583.11 ± 7.10 a
	T1	31.52 ± 2.97 a	34.08 ± 3.00 a	28.70 ± 3.03 b	22.89 ± 2.36 ab	111.89 ± 3.89 ab	3.12 ± 0.07 ab	33.33 ± 2.89 ab	563.08 ± 7.50 a
	T2	29.08 ± 3.20 ab	32.35 ± 2.46 ab	26.63 ± 2.94 bc	20.70 ± 3.02 bc	111.67 ± 5.27 ab	3.09 ± 0.33 ab	30.33 ± 5.03 abc	419.08 ± 9.16 b
	T3	29.11 ± 3.66 ab	30.05 ± 3.20 b	25.76 ± 1.27 c	18.86 ± 2.74 cd	108.89 ± 5.60 b	3.05 ± 0.09 ab	27.67 ± 2.52 bc	348.60 ± 9.96 c
	T4	28.04 ± 3.31 b	29.80 ± 2.96 b	22.21 ± 1.37 d	17.31 ± 1.80 d	99.44 ± 8.06 c	2.97 ± 0.22 b	24.67 ± 2.52 c	285.23 ± 10.39 d

*Different letters indicate the significant differences at P ≤ 0.05 among the different treatments at the same time and variety*.

## Discussion

The safety of herbicides on crops may be represented through agronomic traits and yield. Yuan et al. (2013) found that sigma broad at the recommended dose decreased the leaf area and biomass reduction of *R. Isatidis* seedlings. Increasing concentrations of fluroxypyr decreased the plant height and leaf area of the foxtail millet (Guo et al., 2018, 2019). The yield of foxtail millet was decreased significantly after a high dose of tribenuron-methyl treatment (Ning et al., 2015). In this research, pyrazosulfuron-methyl inhibited the growth of foxtail millet seedlings, which was reflected by reduced plant height, leaf area, and stem diameter. With the prolongation of time after applying pyrazosulfuron-methyl, the agronomic traits of the foxtail millet showed no obvious recovery, which indicated that pyrazosulfuron-methyl caused irreversible damage to the seedlings. A decline in the grain yield of foxtail millet was obtained following ≥30 g ai ha^−1^ pyrazosulfuron-methyl treatments. Furthermore, the decrease in grain yield of Zhangzagu 10 was higher than that of Jingu 21, indicating that Jingu 21 was slightly stronger than Zhangzagu 10 in mitigating herbicide damage.

Photosynthetic pigments are indicators of plant photosynthetic capacity (Gong et al., 2014) and can absorb and transfer light energy and protect chloroplasts from excessive light damage (Xu et al., 2020). Therefore, we can determine the effect of stress factors on plant photosynthesis by measuring photosynthetic pigment contents. Soares et al. (2020) showed that under glyphosate stress, the total chlorophyll and carotenoid contents of *Solanum lycopersicum* L. leaves were decreased. The chlorophyll a, chlorophyll b, and carotenoid contents of foxtail millet were reduced under low light during the grain-filling stage (Yuan et al., 2017a). Guo et al. (2020) reported that the chlorophyll content of foxtail millet under 2-methyl-4-chlorophenoxyacetic acid (MCPA) stress decreased significantly and was not alleviated with prolonged time after application. In this study, the chlorophyll contents of Zhangzagu 10 and Jingu 21 were significantly higher than that of the CK treatment under the ≥30 g ai ha^−1^ pyrazosulfuron-methyl treatment, and there was no obvious recovery with the progress of the fertility process. The herbicide inhibited the synthesis of chlorophyll in the foxtail millet leaves and hindered the photosynthesis of foxtail millet seedlings. Our results are similar to Guo et al. (2019). Carotenoids are light-filling pigments for plant photosynthesis (Wu, 2008). In this experiment, we found that treatment with pyrazosulfuron-methyl at any dose did not significantly reduce the carotenoid content in the foxtail millet leaves, showing that pyrazosulfuron-methyl did not affect the carotenoid synthesis. Huang et al. (2015) found that sigma broad stress significantly reduced the carotenoid content in foxtail millet leaves, which is different from the results of this study.

In this study, treatment with pyrazosulfuron-methyl decreased the *Pn, Tr*, and *Gs* but increased the *Ci* of foxtail millet leaves. These results suggested that the assimilation ability of mesophyll cells to CO_2_ was reduced. Furthermore, the decrease in *Pn* was accompanied by a decrease in photosynthetic pigment content and an increase in *Ci*, indicating that photosynthetic pigment content and non-stomatal factors jointly affected the *Pn* of foxtail millet leaves. The photosynthetic gas exchange index has the characteristic of “apparent,” while the chlorophyll fluorescence parameter reflects the “intrinsic” nature of the leaf light system. Kasajima (2017) found that the treatment of leaves with methyl viologen caused a differential decrease in the *Fv/Fm* of different varieties of rice leaves. Yuan et al. (2017b) reported that treatment with sigma at 3.37 g ha^−1^ significantly reduced the Y(II) and ETR of foxtail millet and inhibited the PSII activity of foxtail millet. In this study, we found that treatment with pyrazosulfuron-methyl reduced the *Fv/Fm*, ETR, and *qP* and increased the *qN* of foxtail millet leaves, indicating that the PSII reaction center was injured and that the leaf thylakoid membrane was damaged. With the prolongation of the time after application, 15 DAT with pyrazosulfuron-methyl ≥30 g ai ha^−1^, photosynthetic gas exchange parameters and chlorophyll fluorescence parameters did not recover significantly, indicating that pyrazosulfuron-methyl caused photoinhibition and photodamage to PSII in foxtail millet seedlings.

Under adverse stress (herbicide), the accumulation of ROS in plant cells results in membrane lipid peroxidation and MDA formation (Jalal et al., 2020). The MDA content can reflect the degree of damage to the plant cell membrane and ROS. Under herbicide stress, the MDA content of maize (Panfili et al., 2019), wheat (Bao, 2020), and soybean (Li et al., 2020) was significantly increased. These results showed a significant increase in MDA concentration of foxtail millet under ≥30 g ai ha^−1^ pyrazosulfuron-methyl treatment, which indicated that the cell integrity of the foxtail millet leaves was compromised; however, with the progress of the fertility process, pyrazosulfuron-methyl at low doses (≤60 g ai ha^−1^) had a certain degree of relief on membrane damage, while there was no obvious recovery under 120 g ai ha^−1^ pyrazosulfuron-methyl treatment.

There are many protective enzymes that protect plants against the damaging effects of ROS, such as SOD, POD, CAT, APX, and GR (Caverzan et al., 2019). Under halosulfuron-methyl stress, the SOD, POD, APX, and GR activities of soybean increased (Li et al., 2020), and the SOD and POD activities of foxtail millet leaves also increased but under broad sigma stress (Huang et al., 2015). Bao (2020) found that the antioxidant system of wheat seedlings responded strongly to imazethapyr, specifically, the activities of SOD, CAT, and GPX enzymes were all significantly induced. Hassan and Nemat Alla (2020) reported that a malfunction to the defense system was induced in wheat by isoproturon resulting in inhibitions in GSH-associated enzymes, and the magnitude of inhibition was most pronounced in GPX followed by γ-glutamyl-cysteine synthetase, glutathione synthetase, glutathione S-transferase, and GR. In this experiment, we found that treatment with pyrazosulfuron-methyl increased the activity of SOD, POD, CAT, APX, and GR in foxtail millet leaves. This result showed that under herbicide stress, foxtail millet seedlings could stimulate the active oxygen scavenging enzyme system, effectively removing excessive active oxygen and membrane lipid peroxidation products in the cell, and protecting the integrity of cells and the dynamic balance of active oxygen. Pyrazosulfuron-methyl at high doses (≥60 g ai ha^−1^) decreased the antioxidant enzyme activity in the foxtail millet leaves, which revealed that the balance of ROS production and scavenging was destroyed and that oxygen stress occurred. Over time, the activities of SOD, POD, CAT, APX, and GR in foxtail millet leaves decreased after pyrazosulfuron-methyl treatment. These results suggested that the effect of the herbicide on the foxtail millet antioxidant enzyme system gradually decreased 15 DAT.

The small-molecule antioxidants in plants include GSH, AsA, tocopherol, and vitamin E (Galant et al., 2011), among which AsA and GSH are important electron donors in the active oxygen non-enzymatic defense system and can participate in the AsA-GSH cycle to remove H_2_O_2_. Nemat Alla and Hassan (2014) found that the isoproturon can significantly decrease the contents of GSH and AsA. Under imazethapyr stress, the GSH content of wheat initially increased but then decreased Bao (2020). Wang H. C. et al. (2018) reported that bispyribac-sodium caused the perturbation of glutathione homeostasis in rice, including the decreased reduced glutathione (GSH) and increased oxidized glutathione (GSSG). In this study, the contents of AsA, DHA, total AsA, GSH, GSSG, and total GSH in foxtail millet leaves were increased under pyrazosulfuron-methyl stress; the circulation efficiency of AsA-GSH was improved; ROS in foxtail millet cells were effectively eliminated. The antioxidant content of millet leaves did not recover to the CK level with prolonged time, which indicated that the injury of pyrazosulfuron-methyl to foxtail millet was not completely alleviated after 15 days. AsA/DHA and AsA/total ascorbate of *Hordeum vulgare* L were significantly decreased under glyphosate stress, while DHA/total ascorbate was significantly increased (Spormann et al., 2019). This was different from our results, probably because of the difference between the two crops.

## Conclusion

In conclusion, the recommended dose of pyrazosulfuron-methyl (30 g ai ha^−1^) is unsafe for foxtail millet. After pyrazosulfuron-methyl (30 g ai ha^−1^) application, the growth and development of foxtail millet were inhibited and the chlorophyll content, photosynthetic rate, and activity of PSII were reduced. At the same time, it decreased the production and removal balance of active oxygen in the foxtail millet. With the extension of time, the damage of pyrazosulfuron-methyl on the foxtail millet was not recovered and ultimately resulted in the reduced yield of the foxtail millet.

## Data Availability Statement

The original contributions presented in the study are included in the article/supplementary materials, further inquiries can be directed to the corresponding authors.

## Author Contributions

XY and LZ designed the experiment. KM and WZ collected the samples, analyzed the samples, and drafted the manuscript. XH and YF performed parts of the experiments in the laboratory. SA revised the manuscript. All authors have read and approved the final manuscript.

## Conflict of Interest

The authors declare that the research was conducted in the absence of any commercial or financial relationships that could be construed as a potential conflict of interest.

## Publisher's Note

All claims expressed in this article are solely those of the authors and do not necessarily represent those of their affiliated organizations, or those of the publisher, the editors and the reviewers. Any product that may be evaluated in this article, or claim that may be made by its manufacturer, is not guaranteed or endorsed by the publisher.
